# An Ultrasonic Tomography System for the Inspection of Columns in Architectural Heritage

**DOI:** 10.3390/s22176646

**Published:** 2022-09-02

**Authors:** Sofía Aparicio Secanellas, Juan Carlos Liébana Gallego, Guillermo Anaya Catalán, Rodrigo Martín Navarro, Javier Ortega Heras, Miguel Ángel García Izquierdo, Margarita González Hernández, José Javier Anaya Velayos

**Affiliations:** 1 Institute of Physical and Information Technologies Leonardo Torres Quevedo (ITEFI), CSIC, C/Serrano 114, 28006 Madrid, Spain; 2 Information Processing and Telecommunications Centre, Av. Complutense 30, 28040 Madrid, Spain

**Keywords:** ultrasonic tomography, cultural heritage, automation, signal processing

## Abstract

A new versatile and geometrically reconfigurable ultrasonic tomography system (UTS) has been designed to inspect and obtain information about the internal structure and inner damage of columns in heritage buildings. This nondestructive system is considered innovative because it aims to overcome common limitations of existing systems. Tomographic inspections are typically carried out manually and are thus limited to small portions of construction elements. The proposed UTS allows the automatization of the inspection and the generation of numerous tomographic slices along the height of the column. It is valid for multiple types of columns and materials. In the present work, the system was tested on two limestone columns of the north façade of the Convent of Carmo in Lisbon, Portugal. The UTS is composed of a mechanical and an electronic system. The mechanical system consists of four linear motion subsystems mounted in a square setup. A transducer is placed on each of the axes, acting as emitter or receiver of the ultrasonic signals. The mechanical system also includes a guide system to adapt the inspections to the complex geometry of the columns. The electronic system allows the control and the synchronization of the movements and the emission/reception configuration of the four ultrasonic transducers.

## 1. Introduction

The use of nondestructive testing (NDT) for the structural diagnosis of heritage structures is particularly valuable, given the need to avoid other techniques that may damage the structure or to minimize damage to historical buildings [1,2]. Prospecting techniques, e.g., ground-penetrating radar (GPR) or sonic/ultrasonic tomography, which allow inspection of the interior of historical construction elements on-site can provide essential information about the inspected structure, namely: (i) level of inner damage and/or presence of voids [3,4]; (ii) inner morphology [5,6]; or (iii) effectiveness of repair interventions on masonry elements, e.g., grout injections [7,8]. Moreover, such techniques can have important cultural value, as they are able to reveal relevant historical information about the inspected building [4,9,10].

Among the different techniques, ultrasonic methods have been widely used, and in particular, tomographic images have attracted a great attention in medical and industrial applications [11]. In the case of cultural heritage, ultrasonic methods are a powerful tool to analyze the internal structure of historical structures as well as possible deterioration zones and cracks. Two important historical buildings in the Black Sea region were studied by ultrasonic velocity in [12]. Zielińska et al. used ultrasonic tomography by numerical studies and experimental tests for the diagnosis of masonry pillars with and without inclusions [13]. The influence of weathering behavior in two sculptures was studied by comparative ultrasonic measurements along the years in [14]. In [15] the application of integrated 3D diagnostic methods is described: terrestrial laser scanner, close-range photogrammetry and ultrasonic tomography to improve the diagnostic process of the conservation state of the inspected columns. In [16], ultrasonic measurements and tomography images were used to assess damage to marble employing a high number of transducers fixed to marble specimens in the tomography reconstruction. However, conventional tomographic inspection methods are difficult to implement and interpret (requiring high levels of expertise) and are highly time consuming. They are typically carried out manually, and, given the need to collect a great amount of data to carry out the tomographic reconstruction, it becomes an important limitation to inspect large components of the building and one of the main reasons why its use is limited in practice. The main limitations are the limited number of measurements, the inability to inspect the entire structure or sculpture and build 3D tomography, coupling and positioning errors associated with manual measurements, and the time spent on these measures, among others. One of the solutions to these problems is the development of automated measurement systems.

**Figure 1:**
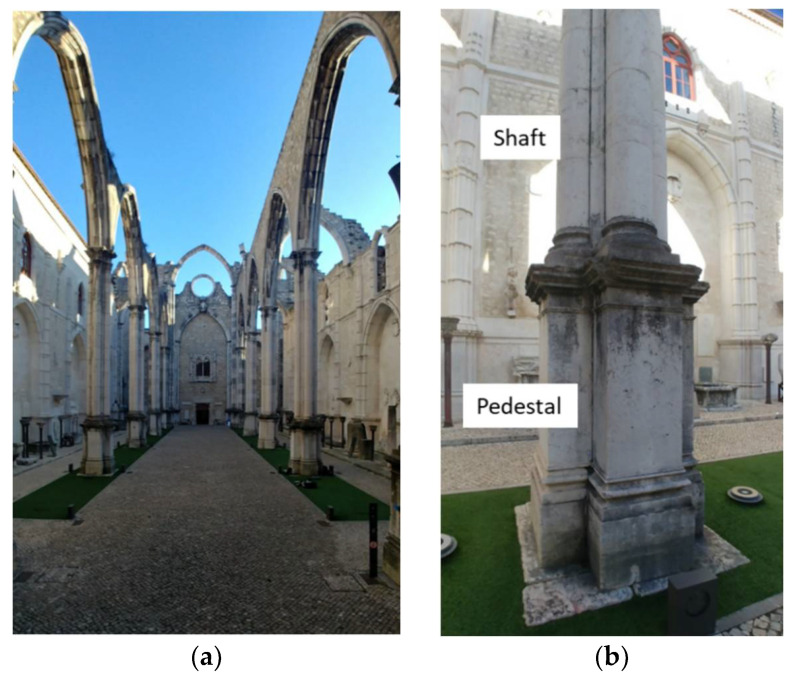
Convent do Carmo (**a**) Central nave (**b**) Column to inspect.

In this work, a new, versatile, and geometrically reconfigurable ultrasonic tomography system (UTS) was designed and developed to characterize the limestone columns of the Convent of Carmo in Lisbon, Portugal (Figure 1). The columns in the convent are made of limestone and they have two different parts: pedestal and shaft. The shafts are composed of multiple drums with a complex geometry.

The tomographic system was used to inspect two columns to a height of 8 m, generating multiple tomographic slices for the generation of 3D tomographic images of the column shafts.

The development of this system was financed by the Heritage Within (HWITHIN) European Research Project of the “Creative Europe” call. The main objective of the HWITHIN project was to introduce a new approach of presenting our built heritage and archeological sites for cultural organizations to innovate at the level of the visiting experience. Specifically, the project mainly focuses on the reconstruction of hidden and unknown features of the architectural and archaeological heritage through the use of new state-of-the-art technologies. The application of these techniques aimed to provide a complete 3D reconstruction of the monument, including not only the exterior surface but also the internal constitution of its constructive elements. Some hidden features that are typically invisible to the naked eye are presented to the visitors in a didactical and interactive way, taking advantage of the potential of augmented reality.

This article is divided into five sections where a detailed description of the ultrasonic tomography system and the methodology used to process the obtained signal are presented. Section 2 introduces a description of the UTS, beginning with previous studies to design the system, and after that, the mechanical and electronic systems are presented. Section 3 explains the methodology used to process the obtained signals with the UTS, and Section 4 shows the results of tomographic inspections. Finally, the conclusions obtained in this work are presented in Section 5.

## 2. Ultrasonic Tomography System

**Figure 2:**
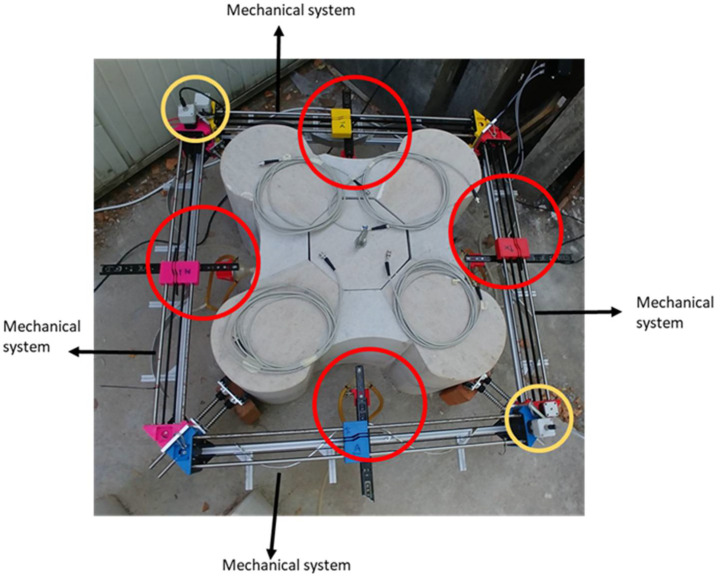
Ultrasonic tomography system developed. Red circle: coupling system with transducer + motion cart and yellow circle: motors.

A new ultrasonic tomography system (UTS) was designed, developed, and fabricated for column inspections in heritage buildings. Although the system was conceived to be used in the columns of the Convent of Carmo, it can be used for any other type of columns and materials. The UTS is composed of a mechanical system and an electronic system. The mechanical system consists of four linear motion subsystems mounted in a square setup (Figure 2). A transducer is placed on each of the axes, acting as emitter or receiver of the ultrasonic signals. The mechanical system also includes a guide system to adapt the inspections to the complex geometry of the columns. The electronic system allows the control and the synchronization of the movements and the emission/reception configuration of the four ultrasonic transducers.

### 2.1. Initial Considerations for the Design of the Ultrasonic Transmission System

To generate images with ultrasound, two methods can be used: pulse-echo and transmission. In this work, the method used was transmission because of the penetration capacity of the ultrasonic waves in the columns under study and because it is easier to interpret the obtained images. In this work, the ultrasonic wave transmitted between the emitter and the receiver transducers if there are no internal reflections is called a ray.

Ultrasonic tomography is a technique that allows the visualization of the cross section, thus enabling better detection of defects, cracks, or discontinuities in the material under inspection. To obtain a tomographic image is necessary to emit and receive multiple ultrasonic rays that cover the whole cross section. A tomographic image is obtained from the velocity or attenuation measurements of the ultrasonic pulses transmitted through the specimen.

To obtain 2D or 3D cross-section image reconstruction is necessary to have a big number of measurements taken from different angles. This requires equipment for the inspections and reconstruction algorithms. Tomography equipment or tomograph consists of multiple ultrasonic transducers placed around the material surface to inspect, an emission-reception system, and an acquisition system. The number of transducers depends on the capability to cover the cross section under study to obtain a high-quality tomographic image. A solution to reduce the number of transducers is moving the sensors around the material, and this solution was adopted in this system. Another aspect to consider is the transmission and reception of ultrasonic waves through the material; this could be contact and noncontact measurements. In contact ultrasonic inspections, the transducer is attached to the material with a coupling agent to minimize the difference in acoustic impedance between air and the surface material. Different couplants can be used: water, glycerine, and medical gel, among others. In noncontact ultrasonic or air-couple inspections, air is used as coupling agent. Although this method is used in the production line, it has lower penetration capacity and is more expensive than contact techniques.

For transmission and reception of ultrasonic waves across the material to be inspected, the contact method with water as couplant was selected in this application. Using water allows ultrasound to pass through microcracked or porous surfaces. This type of couplant has been shown to be useful in determining concrete damage in freeze/thaw cycles [17] and to evaluate the quality of sandstones [18].

**Figure 3:**
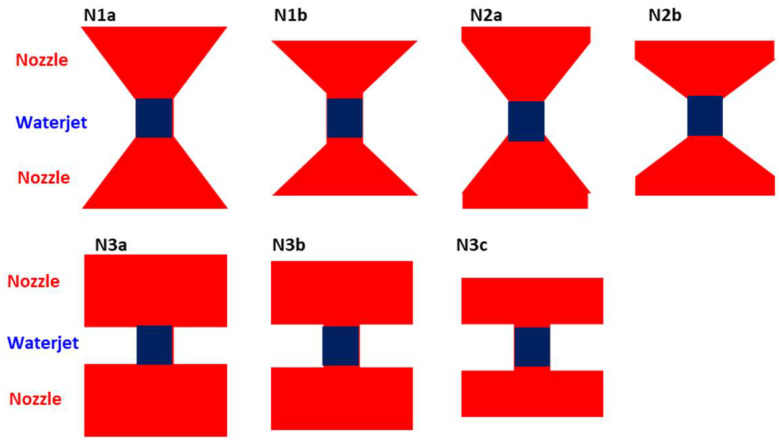
Models (2D) of the nozzles studied with the SimNDT software.

This method is mostly used in automatic testing. The water needs to be preconditioned for obtaining reliable test results, requiring an absence of air bubbles and a minimal particle size. Considering the complex geometry of the columns, a water jet was selected as coupling method. It should be noted that the columns are exposed to the exterior climatic conditions since the convent does not have a roof. Thus, the use of water during the inspection did not put in jeopardy the conservation of the structure. A nozzle was used to guide the waterjet and to mount the ultrasonic transducer. The geometry of the nozzle and the distance of the waterjet between nozzle and stone was studied by simulation of ultrasonic wave propagation using SimNDT software [19,20]. The objective of the study was to determine the optimal geometry to obtain a good ultrasonic image of the interior of the column. For that purpose, several 2D models were studied (Figure 3), considering different geometries and size of nozzles with the diameter and length of the waterjet kept constant (Table 1). The geometry of the nozzle took into account the size of the transducer and the wavelength corresponding to the transducer frequency and the propagation medium, in this case water.

The inspection setup used in simulations was based on transmission mode emitting a longitudinal wave with a 150 kHz five-cycle Gaussian envelope pulse. The transducers had a diameter of 40 mm.

**Figure 4:**
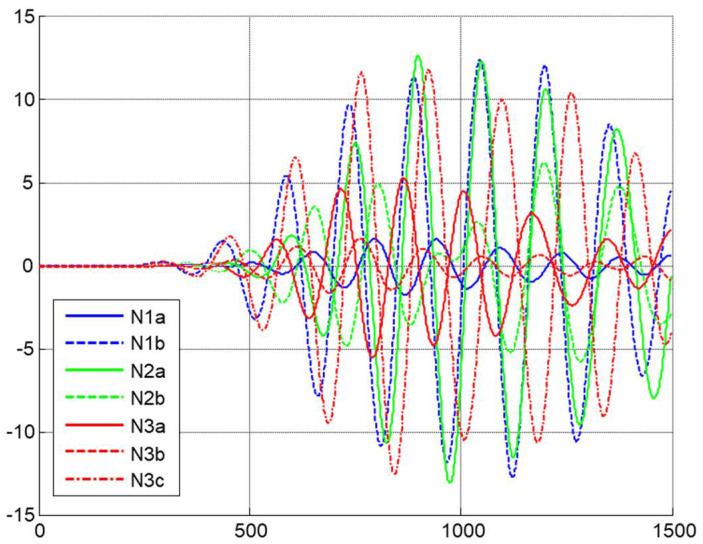
Received A-scans in studied geometries.

Figure 4 presents the received A-scans for the different configurations of the nozzle. To select the appropriate nozzle, it should be considered that the determination of the attenuation and velocity are going to be related to higher amplitude of the receiver signal. Applying this criterion, the most appropriate nozzle was N2a.

**Figure 5:**
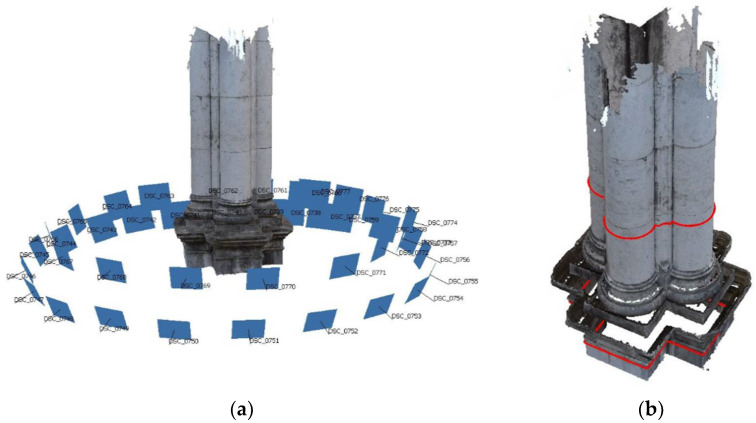
(**a**) Partial photogrammetry of a column performed at the Carmo convent, displaying all locations around the object in blue; (**b**) cross sections of the column shaft and pedestal obtained from the photogrammetry (marked in red).

Other simulations were performed with the selected nozzle geometry and the cross section of the inspected shaft. The drum profile was obtained from the plans supplied by the Convent do Carmo and the photogrammetry made in situ during the inspections, which is a digital technique that allows obtaining geometric information of physical objects based on multiple overlapping photographs (Figure 5).

**Figure 6:**
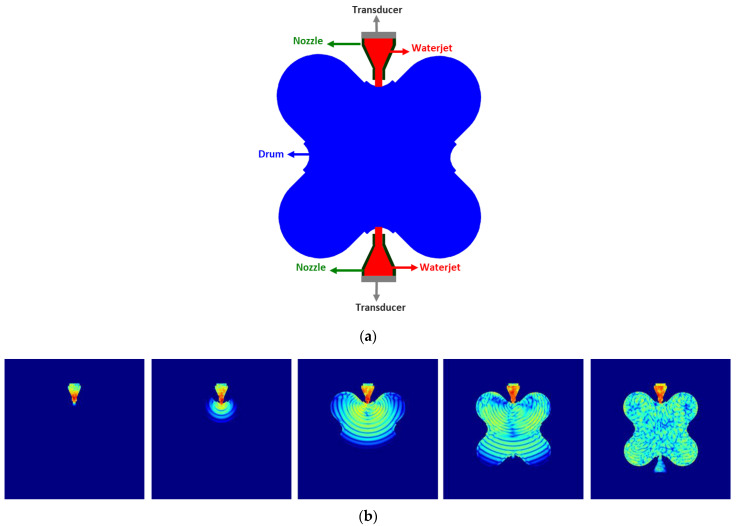
(**a**) Scenario and configurations for simulations; (**b**) snapshots of the wave propagation through the shaft cross section (from video Simulation Nozzle shaft in the Supplementary).

Figure 6 presents the scenario and several snapshots of the wave propagation through the shaft cross section. In the scenario, the nozzle body is modeled as a methacrylate (green) and inside the water (red) while the shaft section is limestone (blue). The inspection setup was the same as used previously. In this case, we considered that the shaft material is limestone. The images were obtained with a dynamic range of 80 dB.

The ultrasonic wave propagates uniformly in water and nozzle, but when it reaches the limestone, the wave front is modified due to the geometry and the amplitude of the wave decreases.

**Figure 7:**
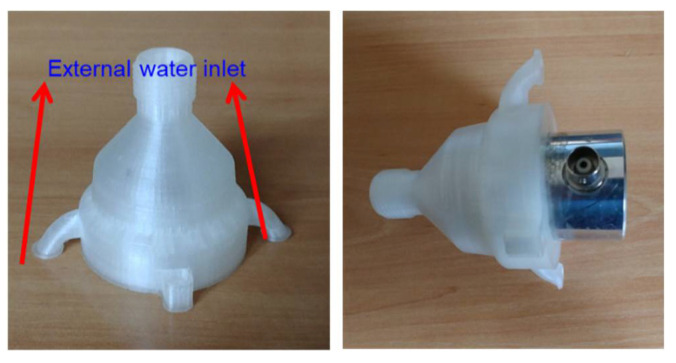
Nozzle used for the UTS.

Therefore, the most adequate geometry of the nozzle after the studies performed by simulation is presented in Figure 7. The designed mechanism consists of a nozzle with two purposes: the ejection of water and the placement of the ultrasonic transducer. This piece was fabricated using a 3D printer.

Before the design of the tomograph, the calculation of geometric parameters that posteriorly will be used to obtain the tomographic image is necessary. For that purpose, several tasks should be performed, namely: (i) calculate the profile of a column section to inspect, (ii) determine the position of emitters and receivers, and (iii) relate each one of the pixels of the column section image with the emitter–receiver ray.

**Figure 8:**
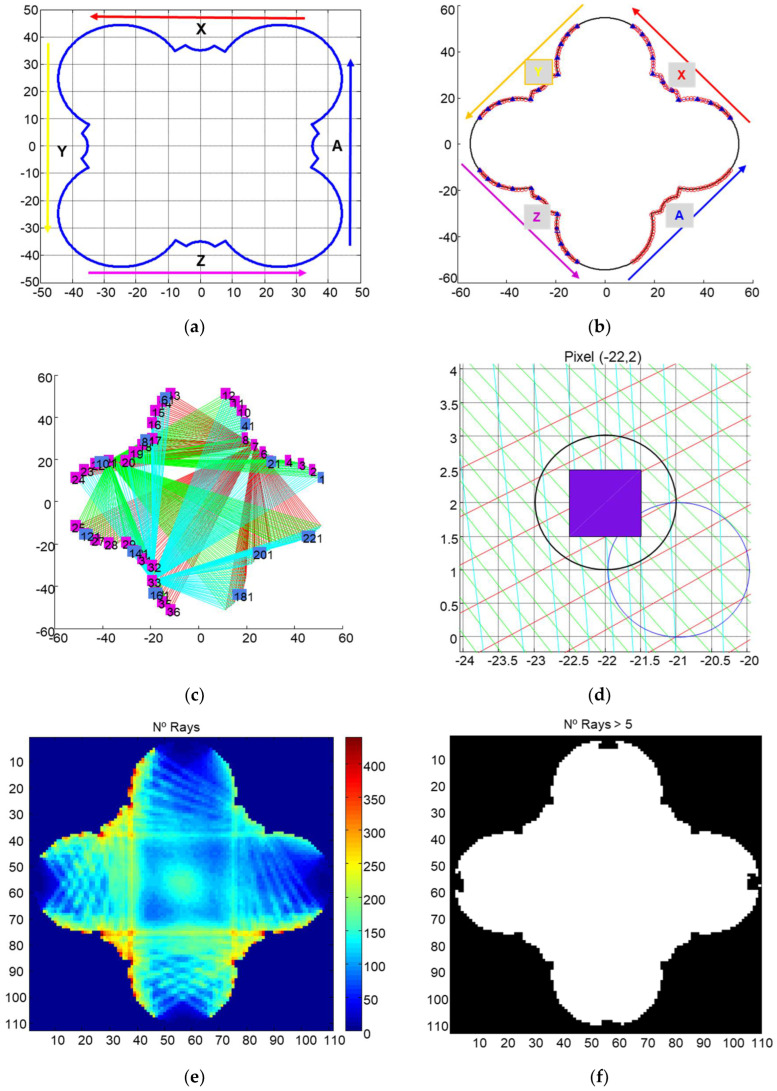
(**a**) Profile of a column cross section with the four axes; (**b**) position of emitters (blue triangles) and receivers (red circles); (**c**) example of generated rays in a cross section; (**d**); schematic representation of projections in a pixel; (**e**) number of rays passing for each pixel; (**f**) pixels with at least 5 rays passing.

Each pixel is equivalent to 1 cm × 1 cm. The position of emitter and receivers depends on the designed system. In this case, there are three axes acting as emitters (X, Y, Z) and four axes acting as receivers (X, Y, Z, A) (Figure 8a). The system has 12 emitters per axis (blue triangles) and 57 receivers per axis (red circles). Figure 8b shows production 6156 rays in total per slice. In Figure 8c, the simulated rays for a tomographic scan are shown. In order to associate the ray with the pixel, first it is assumed that each ray could be represented by the equation of a straight line. The information of each ray is associated with a pixel, but the distance between the pixel and the straight line must be inferior to 1 mm (Figure 8d). The ultrasonic information of the material inspected is associated with the ray passing through it (Figure 8e), and valuable information about reconstruction will be obtained when the number of rays passing through the material is high. Figure 8f shows that at least five rays pass through every pixel within the cross section.

### 2.2. Mechanical System

The mechanical system is formed by several subsystems: the lineal movement, the guide, the lifting, and the acoustic coupling subsystems.

**Figure 9:**
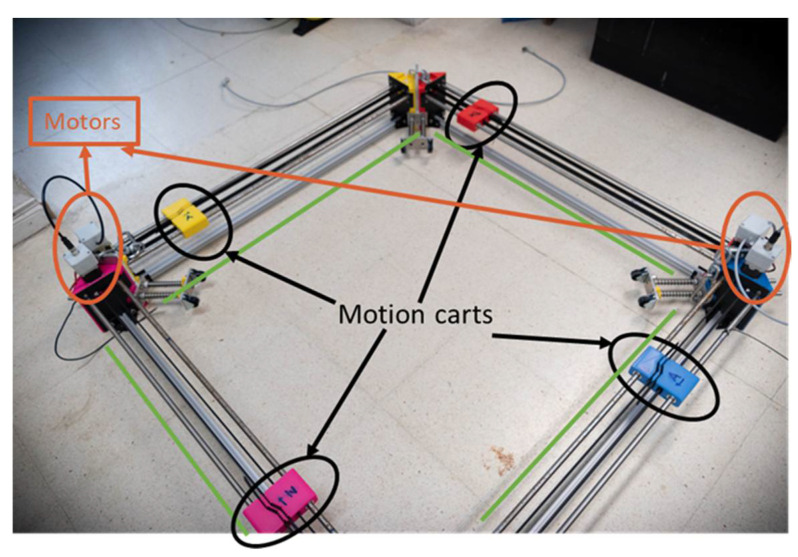
Mechanical system.

The subsystem of linear movement consists of four linear motion axes mounted in a square setup (Figure 9). The four axes are removable and reconfigurable to adapt them to the different geometries of columns. In the case of the convent under study, it adapts to the different shape of pedestals or shafts. A transducer is placed on each of the axes that can act as an emitter or receiver of the ultrasonic waves. A first prototype of this subsystem has been developed previously [21].

**Figure 10:**
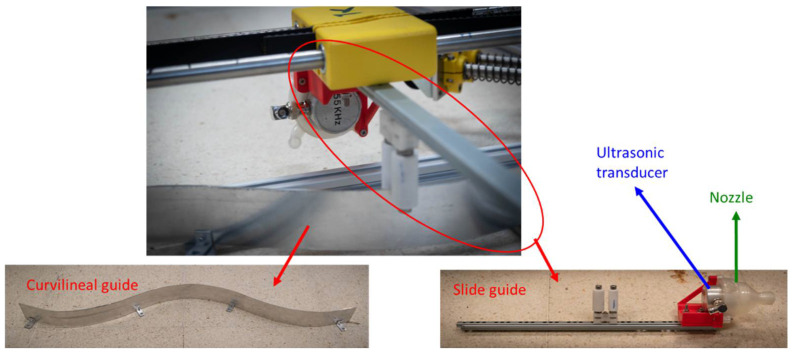
Guide system to inspect the shaft.

A guide subsystem was designed (Figure 10) to adapt the movement of the transducers to the profile of the column and to keep constant the distance between transducer and stone. The system includes a curvilinear guide and a slide guide to follow the curvilinear surface of the shaft. This subsystem allows the sensors to be moved closer or further away from the column as they move along the axes.

**Figure 11:**
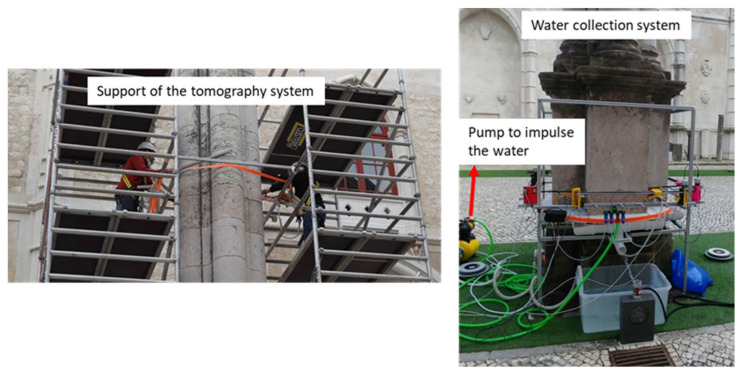
Support of the tomography system on the top of the column, winch to vertical movement, and the system to collect and impulse the water.

The lifting subsystem was designed to precisely position the system to the different shaft heights. It is composed of a platform with four pulleys placed on the top of the column, at 10 m height, and a winch located at the base of the scaffolds (Figure 11). These devices allow positioning the UTS at any height of the shaft.

**Figure 12:**
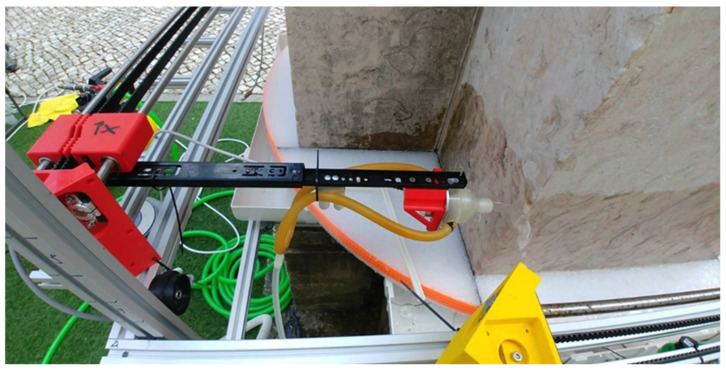
Zoom of the system expelling the waterjet from the pipes to the nozzles and inspecting the base of the column.

In addition to the aforementioned subsystems, a system to collect and recirculate the water and to reduce the consumption of water during the inspections was designed. An impulsion pump, two flexible pipes, and a tank to collect the water form the system. The pump impulses the water from the tank through the flexible pipes to the nozzles (Figure 12). These elements were selected to keep constant the pressure on the nozzles during the inspections. In this system, waterjets come out from the nozzles to the column for each of the four ultrasonic transducers. The water that slides down the column is collected and pumped up to the sensors again, getting a closed water circuit.

### 2.3. Electronic System

The electronic system is responsible for controlling the emission and reception of the ultrasonic waves to generate multiple rays that allow the generation of the tomographic image. For that purpose, the automatic system has to be able to change each axis from emitter to receiver and to vary the movement’s step depending on whether they emit or receive.

**Figure 13:**
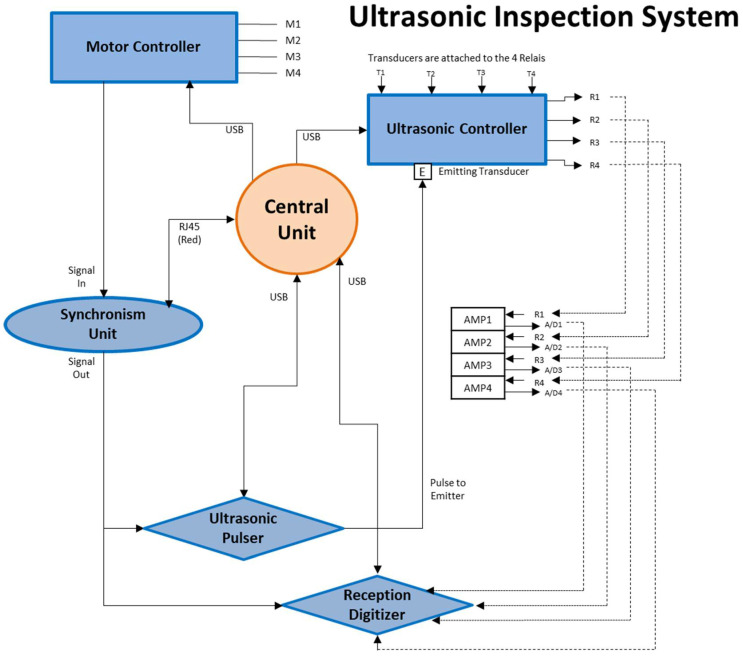
Schematic diagram of connections between the hardware of the ultrasonic inspection system [22].

Figure 13 shows a schematic diagram of the main hardware components that are used in this setup [22].

The hardware components that are part of the automation of the ultrasonic tomography system consist of a central unit in charge of the main program and connecting with the different subsystems (motor and ultrasonic controllers, synchronism unit, ultrasonic pulser and reception digitizer):

(1) Motor controller. This is the system that goes along with the mechanical system of movement around the column. This system comprises an Arduino Uno with a CNC-Shield mounted with 4 stepper motor driver chips mounted on top of it. The stepper motor used has 4 cables connected to it (2 per coil). It can drive 3 independent axes (X, Y, Z) and a slave axis (A). The movement of the A-axis is configured with two jumpers. A circuit board with 2 relays is attached to change the configuration jumpers automatically. Finally another pin on this board is responsible to send a signal to the synchronism unit to initialize the ultrasonic pulse sequence.
(2) Ultrasonic controller. This system is responsible for the configuration of each transducer as transmitter or receiver automatically. Four signal switches are used to make this selection. In this way, each transducer can be connected to a pulse emitter, if it acts as an emitter, or to one of the 4 preamplifiers, if it acts as a receiver. As preamplifiers, the Olympus Model 5662 with 54 dB of gain was used.
(3) Ultrasonic pulser. As a pulse emitter, one based on the IC STHV748S was used. It was programmed with a burst of 5 pulses of 50 kHz and ±100 V amplitude.
(4) Synchronism unit. This system, developed by the authors, will be in charge of the synchronism between the movement of the motors and the ultrasound [23]. The system has a SYNC IN input, a SYNC OUT output, and a network cable through which it connects to the central unit to program the sync sequence. The SYNC IN input comes from the motor controller and the SYNC OUT signal is the one that goes to the ultrasonic pulser and the reception digitizer.
(5) Reception digitizer. The TiePie oscilloscope model HS6diff of four channels was used. It works digitally with a software client on a computer via USB connection. The visualization and the oscilloscope settings can be changed using this desktop client. This oscilloscope will be used to gather the data of the amplified waves of the three receiving transducers.

The sequence of emission and acquisition of ultrasonic signals is as follows. Initially, the transmitting transducer and receivers are selected. When the transducer is placed in the emission position, the motor controller starts the simultaneous movement of the three receiving transducers at a constant speed and activates the SYNC IN signal. When the synchronism unit receives this signal, a digital pulse through SYNC OUT is emitted when the receiving transducers reach the positions that have been programmed. This synchronism signal allows the emitter to generate an ultrasonic pulse and the oscilloscope to digitize the ultrasonic signal received in each of the positions programmed for reception without stopping the movement of these transducers. This sequence is repeated for each of the selected emission positions.

The software that communicates with all the hardware attached to the main computer was developed in Python using a graphical user interface. With this software, we were able to configure the USB ports of the motor and the ultrasonic controllers, the synchronization system, reception digitizer for the reception of the ultrasonic signals, and to send G-code to the CNC-Arduino. The Computer Numerical Control (CNC) works with an open source software library called GRBL that is specifically designed to work with the Arduino Uno + CNC Shield. GRBL is a CNC program, which means that it functions by receiving G-codes and M-Codes. These are codes that describe every movement of the axes, but also pins that are supposed to go to activate spindles or activate cooling mechanisms.

In the automatized program window is where the axes lengths, the number of positions of the emitters, and velocity of the receivers can be changed.

## 3. Processing of the Ultrasonic Tomography Information

The information received by the ultrasonic tomography system described in Section 2 must be processed to obtain the tomographic images of the structure. The methodology to process the data is composed of two steps:

1. Extraction of ultrasonic information from raw captured data
2. Generation of the tomographic image

### 3.1. Extraction of Ultrasonic Information from Raw Captured Data

**Figure 14:**
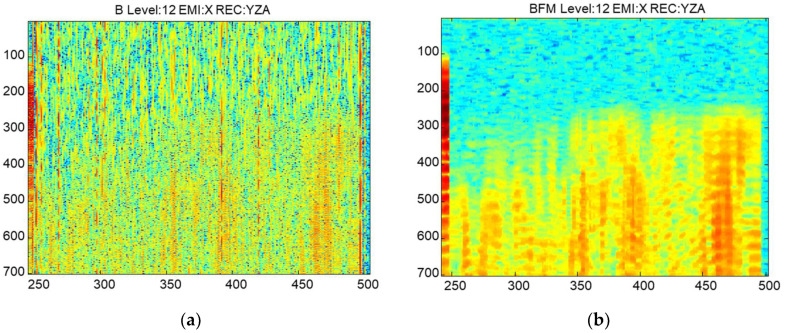
Signal processing. (**a**) Received signal without processing and (**b**) processed. The x and the y-axes represent the emitter and receiver positions, respectively.

To extract the ultrasonic information, several tasks will be necessary to overcome: (i) noise cancellation, (ii) order the acquisitions, (iii) to determine the amplitude and velocity, and (iv) generate the sinograms. The receiver signals have noise that masks the ultrasonic information (Figure 14a). To eliminate this noise, several filters were used: a band-pass filter to remove the electronic and acoustic noise outside the frequency band, an average of the signals to remove impulsive noise, and finally, the envelope of the signals was calculated to improve the stability of the maximum (Figure 14b).

**Figure 15:**
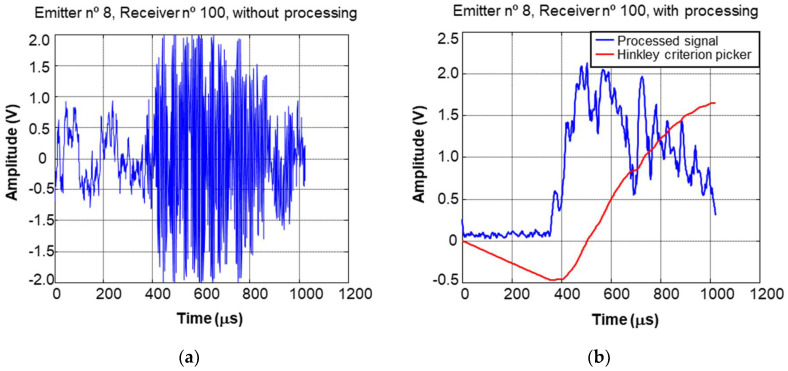
Received A-scan from the cross section of the shaft: (**a**) before and (**b**) after applying a filter.

The determination of amplitude and velocity is made by processing the envelope signal (Figure 15), computing the maximum of the pulse to estimate the amplitude and the beginning of the pulse for the estimation of the propagation time.

Determining the start of the pulse automatically is a complex task. There are multiple factors that make this task difficult. Besides the usual ones, such as acoustic and electronic noise, the coupling used is a significant factor, in this case a waterjet. The water path of the ultrasonic pulse is influenced by the geometry of the column and must be corrected during the processing. Various methods have been tested, and the one that has given the best result is the Hinkley criterion picker, described in [24].

**Figure 16:**
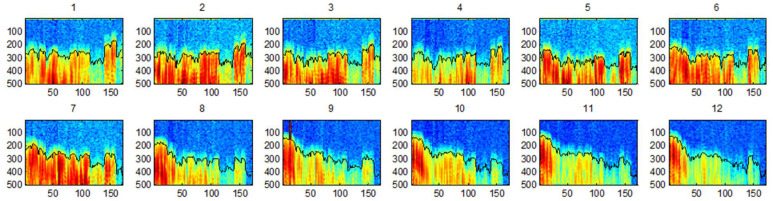
B-scan images from 12 positions of emitters of axis X. The black line represents the automatically detected beginning of the wave.

Therefore, to accomplish this calculation, an approximation has been formulated. From this information, the sinograms were generated. Each point of the sinogram is computed by using the maximum value of the first pulses of the received ultrasonic signal of each A-scan, which corresponds, to the wave front without interference by rebounds on the walls of the column (Figure 16).

**Figure 17:**
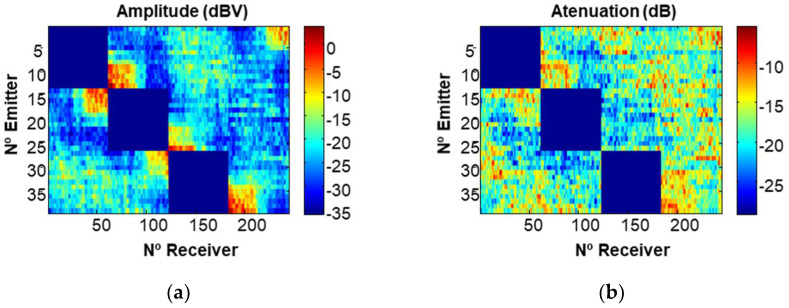
Obtained sinogram: (**a**) amplitude; (**b**) attenuation.

Figure 17 shows an example of an obtained sinogram. In this figure, the x-axis represents the receiver positions (57 receptors × 4 axes= 228) and the y-axis denotes the emitter positions (12 emitters × 3 axes = 36). The zone without amplitude (blue) is due to the fact that the signals of the emitting axis are not received.

Once the amplitude sinogram is obtained, to obtain the attenuation sinogram, it must be divided by a sinogram containing the wave amplitude arriving directly from the transmitter to the receiver in a structure without defects.

### 3.2. Generation of the Tomographic Image

To generate the tomographic images, algebraic reconstruction techniques are going to be employed. These techniques are based on the estimation of the attenuation spatial distribution by the discretization of the inspected scene [25].

From the sinogram, that in this case are composed of 36 emitters and 228 receivers, 36 × 228 = 8208 rays, the tomographic reconstruction methods generate a tomographic image that in this case will be formed by 60 × 60 cells, which are the pixels of the image. As such, the attenuation experienced by a ray that travels through the discretized inspected scene can be written as:

(1) $$p_{m}=\overset{N}{\underset{n=1}{\sum }}a_{m,n}\alpha_{n}$$

where *p_m_* is the attenuation of the *m*th ray, 1 *< m < M =* 8208,$\;\;\alpha_{n}$ is the attenuation associated with the *n*th cell, 1 *< n < N =* 60 × 60, and $a_{m,n}$, is the cell area intersected by the *m*th ray whose path passes through the *n*th cell. Here, it is interesting to note that most of the $a_{m,n}$ coefficients are zero because the path does not pass through them. A tomographic inspection usually consists of thousands of these equations, one per ray, which form a system of linear equations that, for convenience, are expressed here with vector algebra:

(2) p=Aα

where ***p*** = [*p*_1_, *p*_2_, *…*, *p_M_*]^T^ is the vector of measured attenuations, ***α*** = [*α*_1_, *α*_2_, *…*, *α_N_*]^T^ is the vector that contains the discretized values of the attenuation spatial distribution, and **A** is an 8208 × 3600 matrix with generic element *a_m,n_*. Thus, the tomographic reconstruction consists in determining the vector *α* as:

(3) $$\alpha =A^{-1}p$$

This solution implies the inversion of the **A** matrix. However, this is an ill-conditioned and disperse matrix (many rows are linearly dependent and most of the row elements are zero). Thus, solving this system by applying standard procedures, such as direct matrix inversion, least squares, or singular value decomposition, is not possible. Most of the techniques for solving this kind vectorial equation are based on specific iterative methods that are the algebraic reconstruction methods.

**Figure 18:**
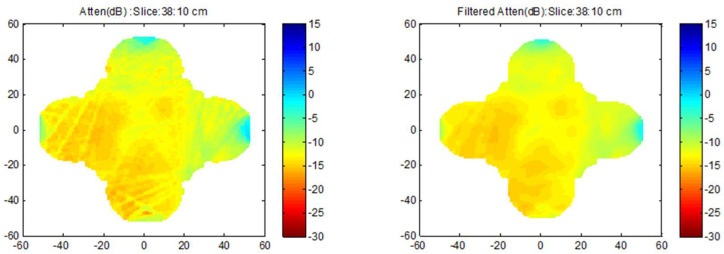
Tomographic reconstruction images before and after smoothing the result obtained considering the attenuation.

Once the tomographic reconstruction of amplitude is obtained, a mean filter (5 × 5 pixels) is performed on the image obtained to smooth the result (Figure 18).

The last step is to generate the 3D image of the interior of the column by means of three-dimensional linear interpolation between the tomographic slices of the dimensions that have been inspected.

## 4. Results Obtained in Convent of Carmo

### 4.1. Case Study: Columns of the Convent Do Carmo

The action proposed the Archaeological Museum of Carmo, in Lisbon (Portugal) as the case study. The museum occupies the ruins of the old church of the Carmo Convent, destroyed during the 1755 Lisbon earthquake. The church was commissioned in 1389 by Nuno Álvares Pereira, the Constable of the Kingdom of Portugal, and was considered one of the most beautiful in Lisbon. The magnificence of the church illustrates the importance of the Carmo brotherhood, which, at the time of the erection of the church, were owners of almost a third of the Portuguese territory [26]. The significance of the church for the city is also illustrated by the great amount of artwork that is known to decorate the church and chapels before its collapse.

The building is located atop the Carmo hill, overlooking the city. The unstable soil condition, mainly composed of weak sands and clays, led to important structural stabilization issues and soil settlements that compromised the safety of the building. Some of the cracks that opened at that time are still visible today. Eventually, the solution was to build flying buttresses to reinforce the southern façade of the church. The church was completed in 1423, but it suffered many transformations along its history. For instance, currently only one flying buttress remains. To maintain the resistant force without the arches, the walls were thickened. Nevertheless, the most significant one occurred after the earthquake of 1755, which led to the almost complete collapse of the building.

Only the chevet area (except for the main chapel vault), part of the main façade and parts of the north and south façade survived the 1755 earthquake. Moreover, a devastating fire followed the earthquake and further damaged the remains and the artwork. Soon after the earthquake, reconstruction works began, trying to follow the model of the original building. However, these were never completed, leading to the current state of partial ruin that is visible today.

**Figure 19:**
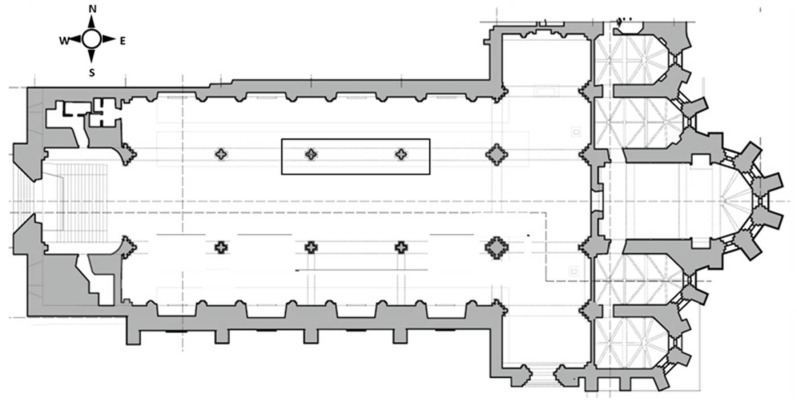
Latin cross plan of Convent do Carmo, highlighting the masonry columns that were inspected with a black rectangle.

The church has a Latin cross-plan configuration with three naves (Figure 19). The original vaulting ceiling of the naves was probably composed of gothic ribbed cross-vaults, similar to the still-remaining vaults of four apse chapels. The main façade also maintains most of the original work and late Gothic decorative features, including the old rose window [26].

The structural elements that were investigated using the novel UTS were the masonry columns of the central nave (Figure 1). They were selected to better understand how they were precisely constructed, since there is insufficient information on the interior morphology of their cross section. Additionally, finding relatable examples to compare to the columns at the Carmo church are quite minimal, making it complicated for even initial assumptions to be established.

The original columns, dating from the Gothic ara, could probably be a compound pier type composed of a bundle of weaker shafts joined as a group to work together while depicting a strong and thin column [27]. The multiple shafts could be composed of solid stone or established through a system of multiple elements. Typically, a compound pier contains a rubble masonry core surrounded by the shafts, conveying the appearance of multiple columns grouped together while still providing structural support to the arches built above. Additionally, having the columns built with this construction technique would create more ease and lighter weight to carry for the laymen who were at work [28]. However, the nave columns were reconstructed after the 1755 earthquake, in a type of neo-Gothic style that was followed during the rebuilding works. Being reerected between 1756 and 1834, the techniques, art style, and means had drastically changed. The inspections using the UTS can shed light on this aspect and help us understand how the columns were precisely rebuilt after the earthquake, as well as showing their state of deterioration along its height.

### 4.2. Results Obtained in Convent of Carmo

The building elements to inspect by ultrasonic tomography were columns 2 and 3 of the north façade of the Convent of Carmo (Figure 1). In these elements, the pedestal and the shafts were inspected. For the installation and inspection of shafts two scaffolds were installed.

**Figure 20:**
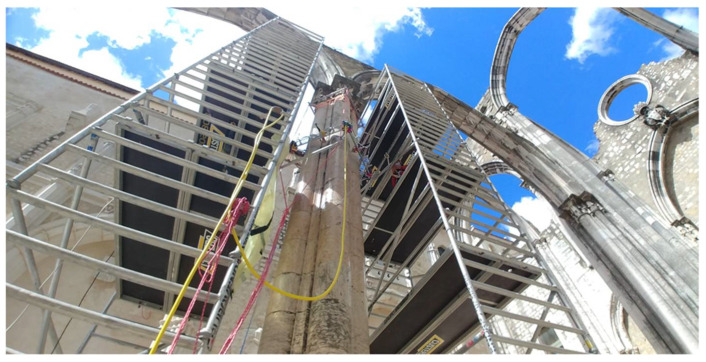
Tomography system inspecting the shaft.

Two experimental campaigns were done to inspect the columns of the Convent of Carmo. The first campaign was performed to inspect the shaft of column 2 from 2 to 12 November 2020. The second campaign was carried out from 26 to 30 April 2021 and the shaft of column 3 and the pedestal of column 2 were inspected. An image of the tomography system during an inspection of the shaft in the Convent do Carmo is shown in Figure 20. It is noted that the installation of the UTS on the shaft of the column 3 took approximately 5 h in the second campaign. The installation on the pedestal of column 2 took approximately 2–3 h.

**Figure 21:**
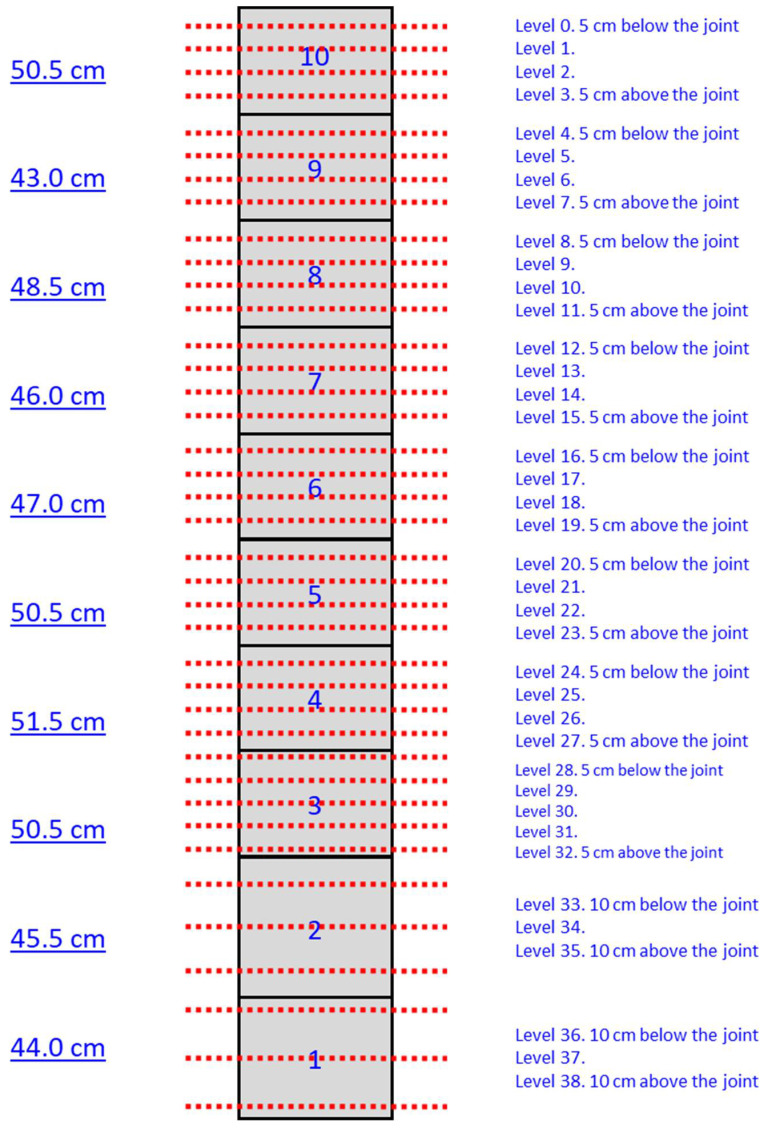
Scheme of the inspections made in shaft of column 3.

For each inspected column, many tomographic slices were done at different heights, and the positions of the slices corresponding to column 2 are shown in Figure 21. Column 3 has 6.71 m of height and 14 drums, but the inspections were done on the first 13 drums (4.77 m), beginning from the pedestal. For each drum, four inspection slices were made, except for drum 11, where 5 inspections were made. The inspection time required for each slice was approximately 5 min.

**Figure 22:**
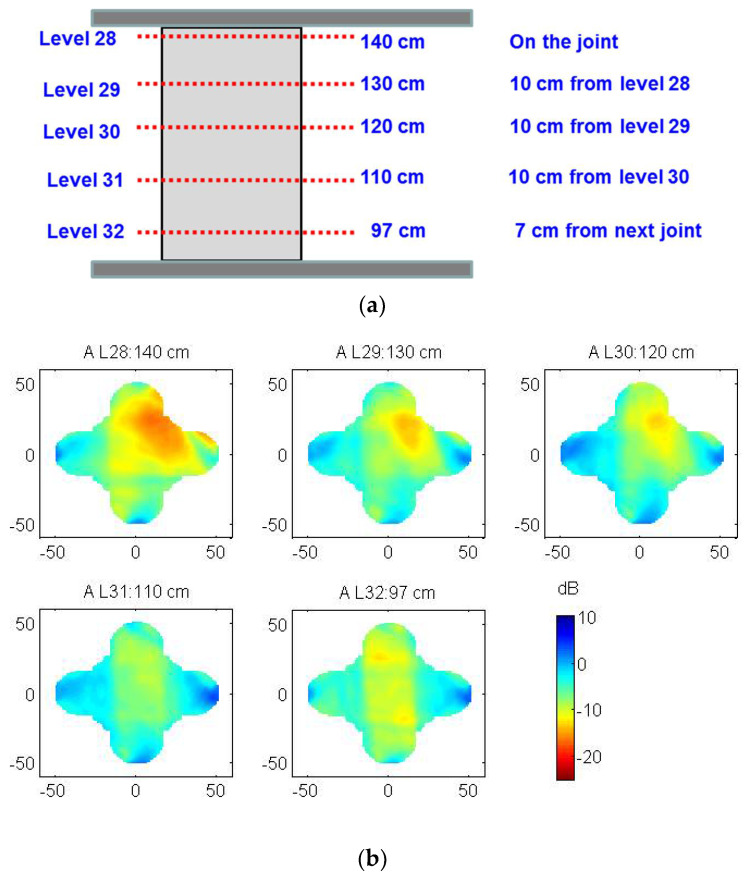
Tomographic images of drum 3 from column 3: (**a**) vertical section of the drum marking the location of the slices; (**b**) tomographic images of the slices.

In Figure 22, the tomographic images of drum 3 from column 3 are presented. The images show, in false color, the wave attenuation in the different slices. The bluish colors represent low attenuation values indicating a uniform homogeneous material, while the reddish colors are high attenuation values indicating the presence of defects or voids in the stones. The smeared red colors that appear on the top left part of the cross section highlight the presence of an anomaly. The red colors progressively disappear towards the bottom slices of the drum. In this drum, a surface crack in the stone could be observed with the naked eye, but the tomographic images confirm that the crack is not superficial and extends through the material. The depth of the crack (which seems to exceed 10 cm at the top part of the drum), as well as its geometry, can be assessed with the tomography results.

**Figure 23:**
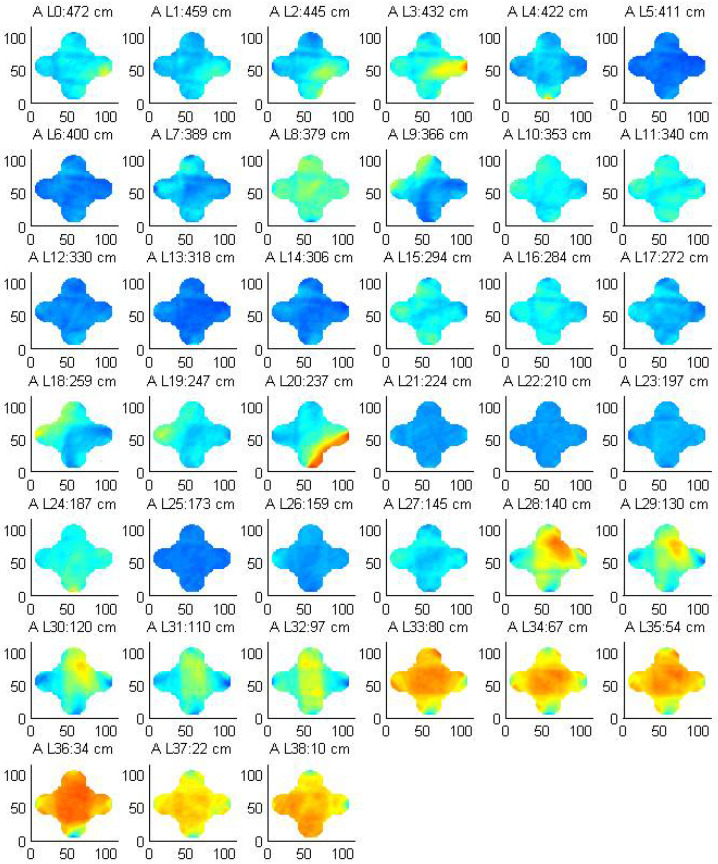
Tomographic images of all drums from column 3.

All tomographic images of the column 3 are presented in Figure 23. The images are ordered (left to right and bottom to top) from highest to lowest. It can be appreciated that the inferior drums have higher attenuation (red colors throughout the whole cross section), which indicates that these stones are more degraded than the higher drums. Assuming that the material is the same along the height of the column (i.e., all stones belong to the same quarry), the high attenuation values obtained throughout the whole cross section of the two bottom drums seem to indicate a higher level of general deterioration of these stones. Indeed, a widespread superficial microcracking could be observed at these stones. Possibly, these stones are more exposed to weathering due to the accumulation of water at the pedestal or they are more porous and thus more prone to deterioration.

It should be noted that the pattern observed in Figure 22 is different from the one observed at the two bottom drums in Figure 23. Local high attenuation values thus typically indicate local anomalies or damage (e.g., cracks) while general high attenuation values throughout the cross section may indicate a widespread deterioration phenomenon occurring at the material. The upper stones show significantly lower attenuation values, but other local defects can be observed mostly occurring at the surface. For example, another anomaly can be detected at the top drum (L3: 432 cm high and progressing upwards). On visual inspection, a past repair was detected. A patch was probably added to repair some local damage on the stone. The ultrasonic tomography is sensitive to these anomalies and reveals that the previous damage was not only superficial, but extended several centimeters deep through the stone. At mid-height (e.g., L20: 237 cm high), high levels of superficial attenuation are observed, which are possibly related to superficial damage at that area.

Nevertheless, the overall uniform low level of attenuation obtained in most drums reveal an overall good state of conservation. Also, this uniform attenuation distribution at the cross section reveals that there are not any interior joints in the column. Therefore, these images seem to indicate that the drums of the columns seem to be composed of single blocks.

**Figure 24:**
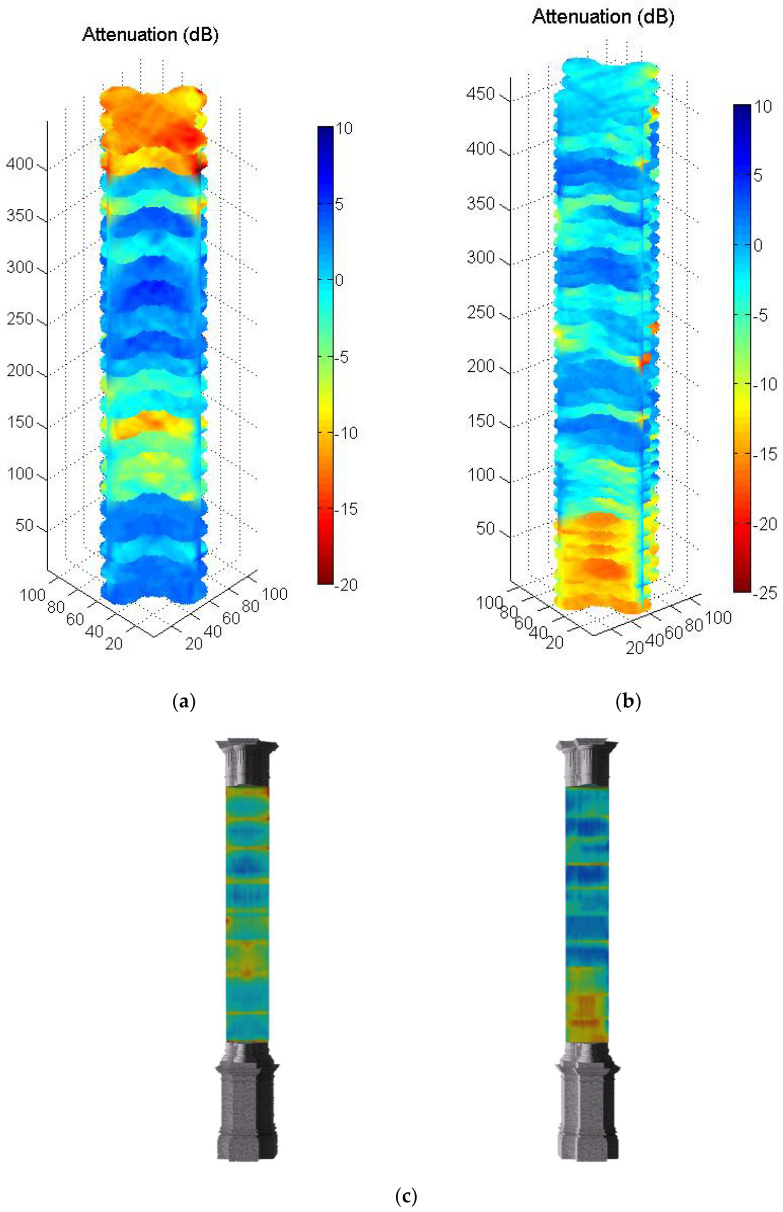
(**a**) Tomographic images (3D) of drums from: column 2, (**b**) column 3, (**c**) integration of tomographic images in a geometrical model.

Another way to present the information is as a 3D element. In Figure 24a,b, a 3D representation of the shaft of the two inspected columns is shown. The integration of the data into a 3D geometrical model enables an easy-on-the-eye visualization and to relate the visible with the non-visible, as well as to look beyond the surface of the object (Figure 24c), which can facilitate the identification of decay phenomena, such as cracks. Such anapproach allows a more immersive and integrated visualization of inspection results that may help technicians and experts in the interpretation. As an example, Figure 24c shows more clearly the location of the most degraded stones (e.g., at the bottom in the case of column 3) and allows locating local damage and their extension in depth (e.g., cracks). It should be noted that these models could be added to visualizing 3D platforms for PC or mobile phone, allowing an easy navigation for the interpretation of the results on-site or remotely.

## 5. Conclusions

In this paper, a novel ultrasonic tomography system (UTS) for columns of heritage structures, designed by the authors, has been presented. The ultrasonic system is composed of a mechanical and an electronic system and aims to overcome typical limitations of conventional tomographic inspection methods. More specifically, the proposed UTS allows to automate the inspection and to perform numerous tomographic slices along the height of the column.

This paper has presented the development and design of the proposed system and its application in a real case study to characterize the limestone columns of the Convent of Carmo in Lisbon, Portugal. Nevertheless, this system is versatile and geometrically reconfigurable and could be used to inspect any column with varying geometry and material. A methodology to process the ultrasonic information of the interior of the structure inspected is also proposed.

The UTS proved to be an efficient tool to inspect heritage structures. In terms of time, after the installation of the system on the structure (approximately one day in a complex structure, such as the columns of the Convent of Carmo), the inspection time required to perform a tomographic inspection of one slice is approximately 5 min. This allows collecting a great amount of data per day and building 3D tomographic images of entire columns. It is noted that tomographic inspections are conventionally carried out manually, which can take up to 3 h to perform an inspection of a single slice and highly reducing the number of emission and reception points.

The results obtained have shown that this system is suitable to obtain valuable information of internal cracks and overall degradation state of the inspected drums of the columns. Moreover, the results also allowed understanding of how the columns were precisely constructed, showing that the shaft is constituted by single blocks of limestone.

Finally, this paper has also shown the potential of integrating the tomographic images into 3D geometric models of the inspected structure. This approach can facilitate the interpretation of complex data that demands high expertise. The easy-on-the-eye visualization of the data in 3D platforms allows us to relate the visible (exterior surfaces) with the non-visible (interior of the element) and precisely locate anomalies and damaged areas. The outcomes of the present research are expected to contribute to the conservation of cultural heritage buildings by offering novel tools and methodologies.

## Figures and Tables

**Table 1:** Size of studied nozzles by simulation.

	Nozzle			Waterjet	
	Dsup (mm)	Dinf (mm)	H (mm)	D (mm)	H (mm)
N1a (λ)	40	10	20	10	10
N1b (λ/2)	40	10	10	10	10
N2a (λ)	40	10	25	10	10
N2b (λ/2)	40	10	15	10	10
N3a (λ)	40	10	20	10	10
N3b (λ/2)	40	10	15	10	10
N3c (λ/2)	40	10	10	10	10

## Data Availability

Not applicable.

## Supplementary Materials

The following supporting information can be downloaded at: https://www.mdpi.com/article/10.3390/s22176646/s1.
